# Cancer stemness-associated LINC02475 serves as a novel biomarker for diagnosis and prognosis prediction of hepatocellular carcinoma

**DOI:** 10.3389/fgene.2022.991936

**Published:** 2022-09-02

**Authors:** Xian Lin, Lianxiang Luo, Yujiao Zou, Jian Chen

**Affiliations:** ^1^ Shenzhen Key Laboratory of Inflammatory and Immunology Diseases, Peking University Shenzhen Hospital, Shenzhen Peking University-The Hong Kong University of Science and Technology Medical Center, Shenzhen, China; ^2^ The Marine Biomedical Research Institute, Guangdong Medical University, Zhanjiang, China; ^3^ The Marine Biomedical Research Institute of Guangdong Zhanjiang, Zhanjiang, China; ^4^ Department of Radiation Oncology, Zhujiang Hospital, Southern Medical University, Guangzhou, China

**Keywords:** hepatocellular carcinoma, prognosis, cancer stemness, LINC02475, lncRNAs

## Abstract

**Purpose:** Hepatocellular carcinoma (HCC) is a severe malignant tumor with high incidence and mortality. LncRNAs present broad clinical application prospects. Herein, we aim to identify a cancer stemness associated lncRNA and reveal its role in HCC diagnosis, prognosis evaluation, and progression.

**Methods:** The cancer stemness-associated LINC02475 in HCC samples were identified using bioinformatics analysis. Cellular and molecular experiments were conducted to elucidate the role of LINC02475 in HCC.

**Results:** The firm links between LINC02475 and HCC stemness and prognosis were demonstrated by bioinformatics analysis of public cancer datasets. LINC02475 expression was elevated in HCC, performed well in the diagnosis, and independently predicts poor overall survival (hazard ratio = 1.389, 95% confidence interval = 1.047–1.843, *p* = 0.023), as well as progression-free survival (hazard ratio = 1.396, 95% confidence interval = 1.016–1.917, *p* = 0.040) of HCC patients. Moreover, LINC02475 enhanced the tumorigenic pathways necessary for cell stemness, DNA replication required for cell proliferation, epithelial-mesenchymal transition involved in metastasis, and DNA damage repair pathways that drove cell radioresistance and cisplatin resistance, thus promoting HCC progression.

**Conclusion:** Cancer stemness-associated LINC02475 independently predicted a poor prognosis and promoted HCC progression by enhancing stemness, proliferation, metastasis, radioresistance, and chemoresistance. Our study lays a foundation for the clinical application of LINC02475 as a novel biomarker and target for the diagnosis, prognosis evaluation, as well as treatment of HCC.

## Introduction

Liver cancer ranks among the top three cancer-related mortality worldwide, approximately 90% of which are hepatocellular carcinoma (HCC) (Llovet et al., 2021; Siegel et al., 2022). Surgery and liver transplantation are the preferred strategies for treating HCC with a limited scope of application (Gunasekaran et al., 2021). Transcatheter arterial embolization chemotherapy and targeted therapy are also important methods for HCC treatment with unavoidable therapy resistance (Huang A. et al., 2020). In addition, resistance to radiotherapy and chemotherapy is one of the major factors contributing to poor HCC patients’ prognosis. Cancer stem cells (CSCs) are a subpopulation of cancer cells harboring the capacity for self-renewal and generation of heterogeneous tumors that could induce tumor initiation and lead to relapses (Walcher et al., 2020). It is known that poor sensitivity of HCC stem cells to treatment confers HCC therapy resistance, resulting in recurrence and poor survival of HCC patients (Craig et al., 2020). Therefore, developing novel methods targeting HCC stem cells will improve therapy resistance and the prognosis of HCC patients.

Over the past few decades, considerable efforts have been made to identify biomarkers for the early diagnosis and prognosis of HCC, which were also greatly beneficial to new drugs discovery. For example, a recent study identified serum laminin γ2 monomer as a novel diagnostic and predictive biomarker for HCC (Yamashita et al., 2021). In addition, serum ST6GAL might be a new biomarker that identifies lenvatinib-susceptible FGF19-driven HCC (Myojin et al., 2021). Furthermore, it was reported that Stathmin 1 has potentials for clinical applications as a biomarker for pathological diagnosis and prognostic prediction of HCC (Cai et al., 2022).

LncRNAs are transcripts of more than 200 nucleotides and can be conveniently detected, gradually becoming one of the research hotspots in epigenetics and oncology. Recently, accumulating studies have shown the potential values of lncRNAs in the diagnosis, treatment and prognosis evaluation of HCC (Huang Z. et al., 2020; Li et al., 2020), reflecting their broad clinical application prospects. For example, lncRNA NBR2 is revealed to regulate autophagy of HCC, providing a new target for HCC treatment (Sheng et al., 2021). In addition, it has been reported that lncRNA-PDPK2P promotes HCC progression by modulating the PDK1/AKT/Caspase three signaling pathway, indicating this lncRNA might serve as a molecular target for HCC therapy. Moreover, it was reported that a novel lncRNA NIHCOLE plays a key role in DNA repair and cellular fitness in HCC, which might be a new therapeutic target for HCC treatment (Unfried et al., 2021). Furthermore, increasing number of studies demonstrated that lncRNAs participate in the modulation of HCC stemness and other functions, thus affecting the occurrence and development of HCC (Tang et al., 2020; Statello et al., 2021; Yuan et al., 2021). Therefore, studies focusing on lncRNAs will provide potential strategies for targeting HCC stemness and alleviating HCC progression.

In this work, we aim to select valuable candidate lncRNAs that are involved in the regulation of HCC stemness and are related to HCC patients’ prognosis using bioinformatics analysis based on The Cancer Genome Atlas (TCGA) and International Cancer Genome Consortium (ICGC) database. Interestingly, in the current study, we identified LINC02475 as a potential lncRNA correlated with HCC stemness, whose biological role in HCC has never been reported. In particular, this study also explored the role of LINC02475 in HCC progression and further investigated the potential value of LINC02475 as a biomarker for diagnosis and prognosis evaluation and as a target for HCC therapy.

## Materials and methods

### Data sources and bioinformatics analysis

The RNA-seq and clinicopathological data of Liver hepatocellular carcinoma (LIHC) and Liver Cancer—FR (LICA-FR) datasets were obtained from TCGA (https://www.cancer.gov/tcga) and ICGC database (https://dcc.icgc.org/), respectively. The radioresistance- and platinum drug resistance-associated gene sets were constructed based on a previous study (Jeong et al., 2006), and KEGG websites (https://www.kegg.jp/), respectively. The differential expression analysis, correlation analysis, survival analysis, weighted gene co-expression network analysis (WGCNA), as well as gene set enrichment analysis (GSEA) were carried out as described in our previous study (Lin et al., 2021). Samples were assigned as high and low expression groups on the basis of the best cut-off value for survival analyses in TCGA database.

### Cell culture and transfection

Huh7 and HCCLM3 cells purchased from the Chinese Academy of Sciences Cell Bank with STR verification were cultivated in Dulbecco’s modified Eagle medium (DMEM) containing 10% fetal bovine serum, and cultured under the condition of 37°C with 5% CO_2_. A smart silencer of LINC02475 was obtained from RiboBio Corporation (Guangzhou, China) for knockdown of LINC02475. Briefly, Huh7 and HCCLM3 cells were cultured in 6-well culture plates overnight, then the smart silencer with siRNAs and ASOs were transfected into target cells by employing Lipofectamine™ 2000 purchased from Invitrogen Corporation (Shanghai, China), and cells were further collected and analyzed at 48–72 h after transfection. Cells were routinely detected to be free of *mycoplasma* contamination.

### Quantitative RT-PCR (qPCR) assay

Cells were harvested for total RNA isolation. The reverse transcription was performed to synthesize cDNA as a template. Then qPCR assay was performed with specific primers on Bio-Rad CFX 96 as we described in our previous study (Lin et al., 2022). The relative LINC02475 level was calculated by the 2^−ΔΔCt^ method.

### Tumorsphere formation assay

This assay was previously described in our published work (Lin et al., 2020). In short, cells (5,000 cells/well) were incubated in 6-well ultra-low-attachment plates with serum-free DMEM/F12 containing 20 ng/ml of FGF, 20 ng/ml of EGF, and 2% B27. The number of tumorspheres were recorded and counted for three generations with a 14 days of culture for each generation.

### Immunofluorescence

Cells were seeded on coverslips and then fixed with 4% paraformaldehyde and permeabilized with 0.1% Triton X-100 before incubating with specific antibodies (Supplementary Table S1). Whereafter, cells were photographed under a fluorescence confocal microscope after co-staining with DAPI.

### EdU assay

Cells were managed according to the instructions of EdU Kit (APExBIO Corporation, Huston, United States). A fluorescence microscope (ZEISS, Germany) was utilized for capturing images.

### Transwell assay

Cells suspension were cultured in the upper chamber of transwell treated with (for invasion assay) or without (for migration) Matrigel. The lower chambers of transwell were added with DMEM containing 10% fetal bovine serum. Then, the migrated and invaded cells were fixed, stained, and observed under a microscope.

### Colony-formation assay

Cells were cultured in 6-well plates (200 cells/well) and stimulated with indicated dosages of cisplatin for 6 h or 4 Gy radiation. Then colonies were fixed, stained with hematoxylin, and photographed after culture for indicated days as we previously reported (Chen et al., 2020).

### Statistical analysis

The data were analyzed using RStudio or SPSS 22.0. The variables are presented as the mean ± SD for normal distribution data from at least three independent assays and calculated as the median ± range for non-normal distribution data. The Wilcoxon rank-sum tests and the Student’s t-test for two groups, and one-way ANOVA for multiple groups were adopted for measuring statistical significance. The Spearman’s rank correlation test was adopted for the correlation analyses. Kaplan-Meier survival curves with log-rank tests were plotted for investigating a survival difference. Cox regression models were used to reveal the relationship between LINC02475 and the survival time of HCC patients. A *p* value <0.05 was considered as significant.

## Results

### Identifying the prominent genes correlated with HCC stemness

**FIGURE 1:**
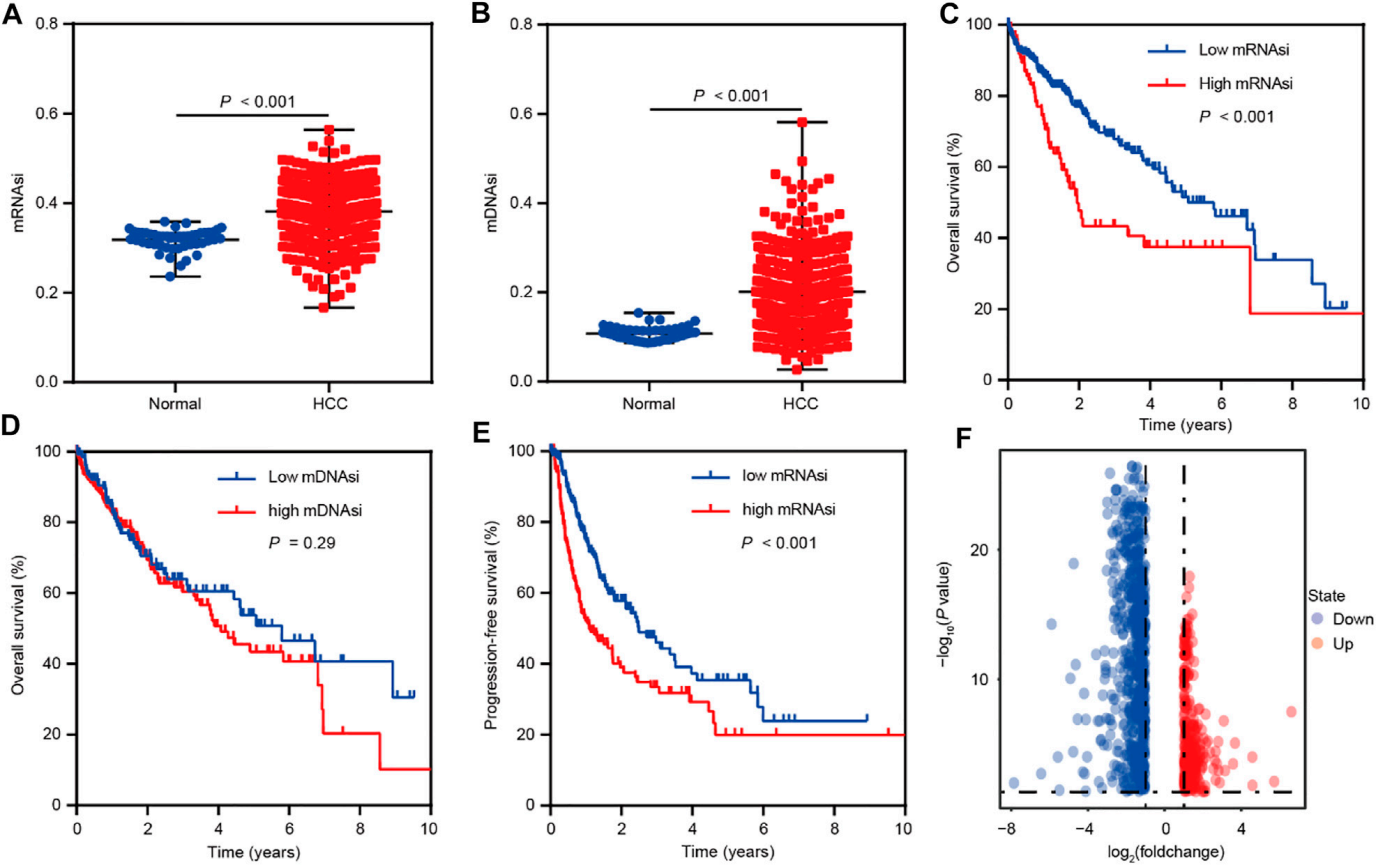
Bioinformatics analyses reveal the role of stemness indexes in HCC as per TCGA database. **(A)** The differential distribution of mRNAsi in HCC and corresponding normal tissues. **(B)** The differential distribution of mDNAsi in HCC and corresponding normal tissues. **(C)** Kaplan-Meier survival curves were plotted to display the overall survival of HCC patients based on mRNAsi values. **(D)** Kaplan-Meier survival curves were plotted to present the overall survival of HCC patients based on mDNAsi values. **(E)** Kaplan-Meier survival curves were plotted to show the progression-free survival of HCC patients based on mRNAsi values. **(F)** The volcano plot of differentially expressed genes between high and low mRNAsi groups assigned based on the cut-off value of survival analysis.

To identify the genes correlated with HCC stemness, we conduct analyses based on mRNA expression-based stemness index (mRNAsi) and DNA methylation-based stemness index (mDNAsi) that have been used as effective indexes in assessing cancer stemness (Malta et al., 2018; Lin et al., 2021). The differential analyses were performed based on the TCGA LIHC dataset and showed that mRNAsi and mDNAsi values were elevated in HCC comparison to normal tissues (Figures 1A,B). Moreover, mRNAsi values were positively related to the poor overall survival of HCC patients, however, mDNAsi values showed no impact on HCC patients’ survival (Figures 1C,D). Therefore, we focused on mRNAsi to conduct further investigation in HCC. Survival analysis also suggested the positive correlation between mRNAsi values and poor progression-free survival of HCC patients (Figure 1E). Confirming the significant role of stemness index (mRNAsi) in HCC, we then conducted differential analyses between high and low mRNAsi groups assigned based on the cut-off value of survival analysis. A total of 1,011 differentially expressed genes (DEGs) were screened out (Fold change ≥2, *p* < 0.05) (Figure 1F), and the correlation analyses further identified 1,004 of these DEGs as mRNAsi-related genes in HCC (*r >* |0.1|, *p* < 0.05).

**FIGURE 2:**
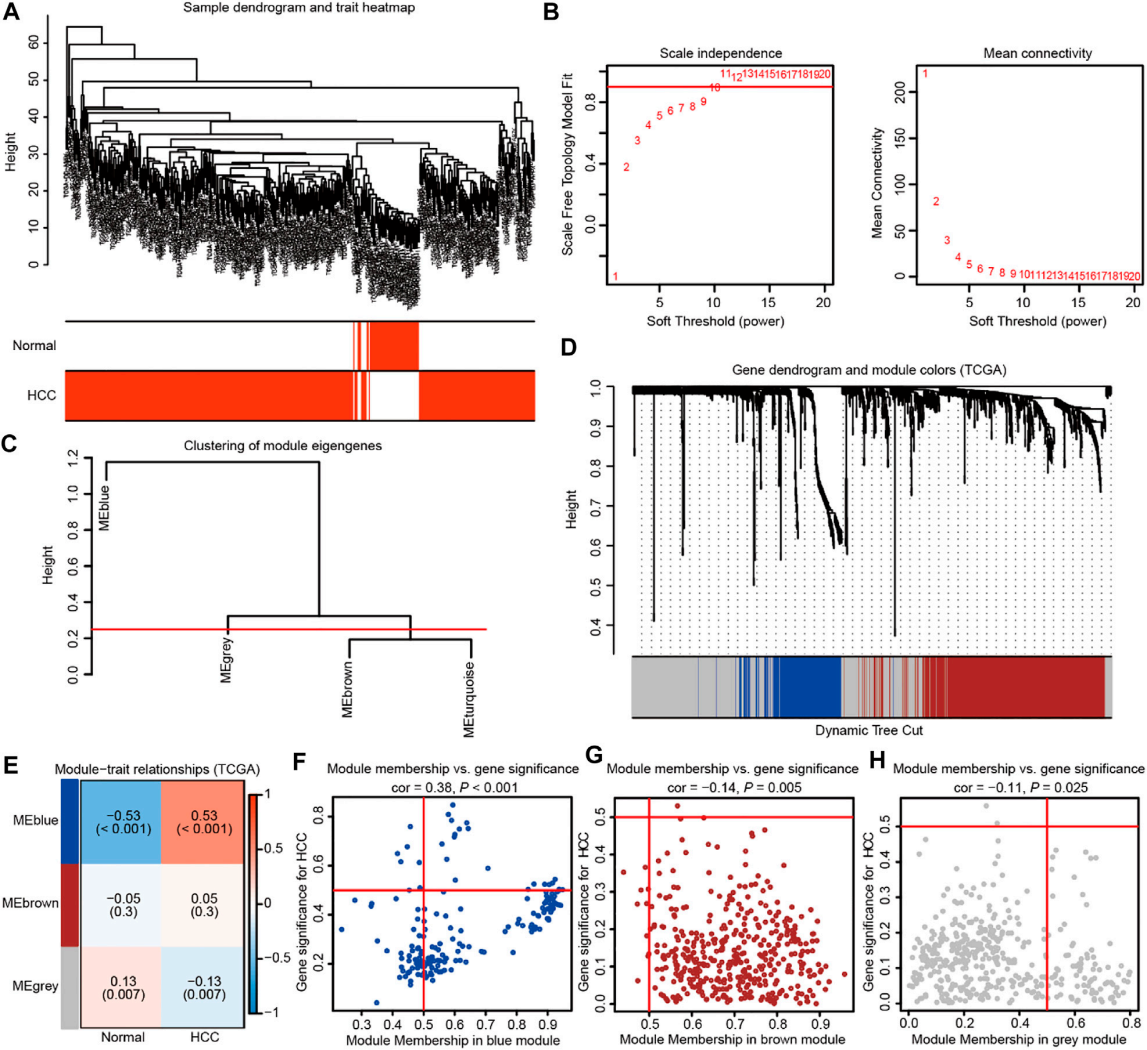
Weighted gene co-expression network analysis identifies the prominent genes correlated with HCC stemness in TCGA database. **(A)** The dendrogram and heatmap showing the distribution of HCC and corresponding normal samples. **(B)** The power value was chosen based on the mean connectivity and scale independence. **(C)** The Gene Tree showing different gene modules according to the power value. **(D)** The cluster dendrogram delineating the three different gene modules as the branches. Each leaf indicated a gene, and each module in a specific color represented a cluster of relevant genes. **(E)**The heatmap showing the correlations and significant differences between the three gene modules and HCC or corresponding normal samples. The correlation coefficient in the upper row and *p* value in the brackets were shown in gene modules, respectively. **(F–H)** The scatter plots exhibiting three gene modules tightly related to HCC samples.

WGCNA was subsequently adopted to investigate mRNAsi-related genes strongly linked to HCC compared with normal tissues. In the process of constructing a gene co-expression network, the dendrogram and heatmap were first plotted to show the distribution of HCC and corresponding normal samples (Figure 2A). Then, gene modules were generated according to the selected power value (Figures 2B,C). Furthermore, the cluster dendrogram was drawn to delineate three different gene modules and clusters of relevant genes (Figure 2D). The correlation between identified modules and HCC was calculated (Figure 2E), and the association between specific genes and HCC was elucidated in the three established gene modules (Figures 2F–H). Therein, the MEblue module was shown to be most strongly related to HCC. The thresholds were defined as *P*_.GS.Tumor_ < 0.001, and 455 mRNAsi-related genes related to HCC were elucidated. Subsequently, GSEA was used to reveal the genesets that these genes participated in. The GO and KEGG enrichment analyses indicated the enriched pathways in HCC progression, including extracellular matrix organization, nuclear division, growth factor binding, cell cycle, PI3K-AKT signaling, focal adhesion, and so on (Supplementary Figures S1A,B). Moreover, the cluster analyses suggested the tight connection of the genesets identified in GO and KEGG enrichment analysis (Supplementary Figures S1C,D). Collectively, these findings suggest the important role of HCC stemness-related genes.

### High LINC02475 expression was observed in HCC and conferred a poor patients’ prognosis

**FIGURE 3:**
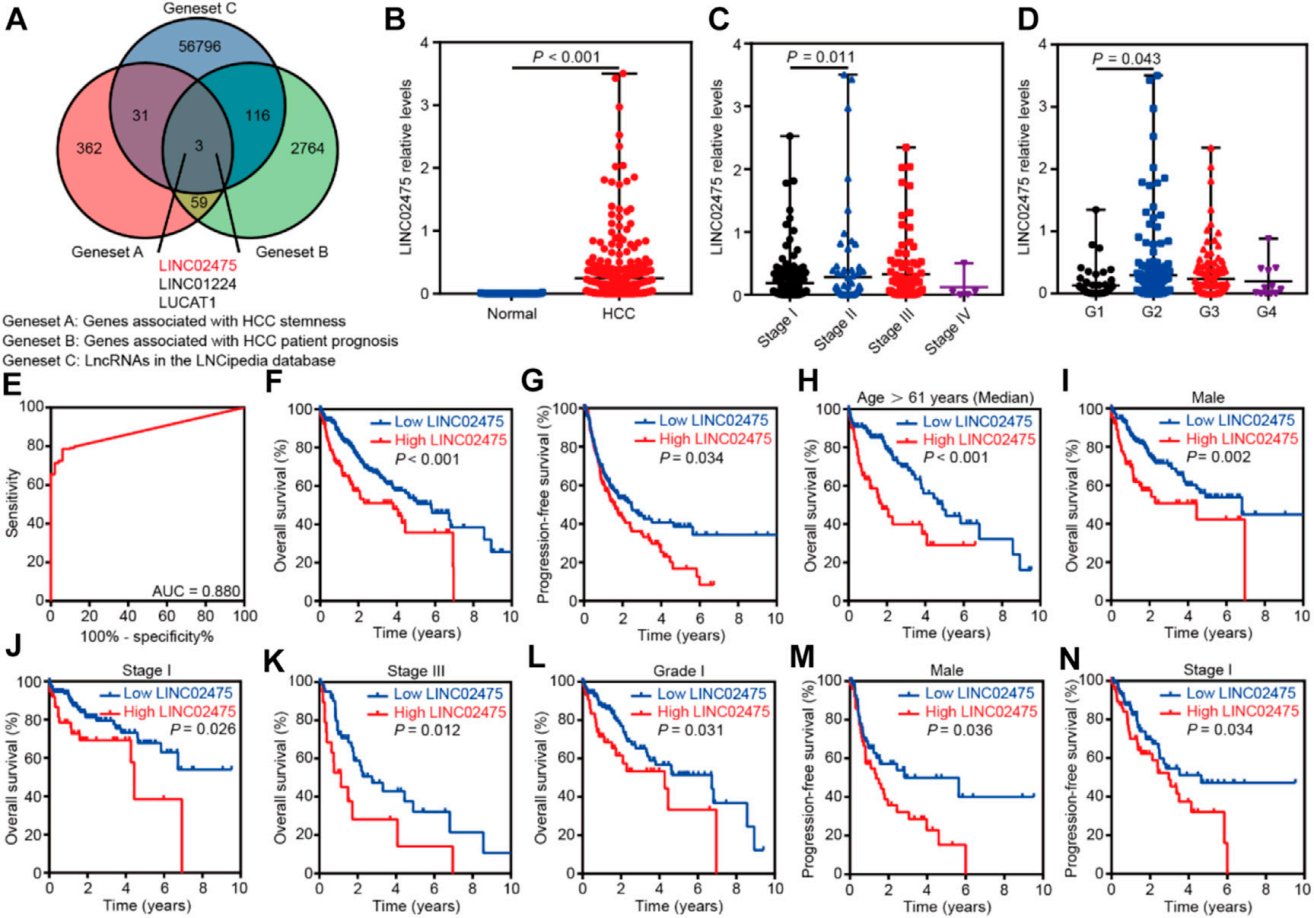
LINC02475 expression was elevated in HCC and conferred a poor patients’ prognosis. **(A)** The Venn diagram showing the overlapping lncRNAs associated with HCC stemness and prognosis. **(B)** The differential distribution of LINC02475 expression between HCC and normal samples. **(C)** The differential distribution of LINC02475 expression in HCC samples with Stage I-IV. **(D)** The differential distribution of LINC02475 expression in HCC samples with grades 1-4. (**E)** The receiver operating characteristic curve was presented to show the prediction role of LINC02475 expression in HCC diagnosis. **(F)** Kaplan-Meier survival curves were plotted to reveal the association between LINC02475 expression and overall survival of HCC patients. **(G)** Kaplan-Meier survival curves were plotted to detect the relationship between LINC02475 expression and progression-free survival of HCC patients. (**H–L**) Kaplan-Meier survival curves of subgroup analyses were plotted to display the overall survival of HCC patients in older (age >61 years) **(H)**, male **(I)**, stage I **(J)**, stage III **(K)**, and grade 1 **(L)**. **(M,N)** Kaplan-Meier survival curves of subgroup analyses were plotted to show the progression-free survival of HCC patients in males **(M)**, and stage I **(N)**.

We compared the genes associated with HCC stemness with those correlated with HCC patient prognosis to identify a specific lncRNA that exerted an important role in HCC. First, we obtained the 455 HCC stemness-related genes elucidated by WGCNA (geneset A). Furthermore, survival analyses showed a correlation between 2,942 genes and the overall survival of HCC patients in the TCGA LIHC dataset (*p* < 0.001) (geneset B). In addition, the 56,946 known lncRNAs in LNCipedia database (https://lncipedia.org/) were obtained (geneset C). A total of three overlapping lncRNAs were got from the intersection of the three genesets (Figure 3A).

Subsequent differential analyses of the TCGA LIHC dataset showed that LINC02475 expression was elevated in HCC compared to that in normal tissues (Figure 3B). Furthermore, high LINC02475 expression was detected in HCC patients with Stage II and grade 2 in comparison to those with Stage I and grade 1, respectively (Figures 3C,D). Interestingly, LINC02475 expression showed high accuracy in the diagnosis of HCC (AUC = 0.880) (Figure 3E). Importantly, survival analyses indicated the positive correlation of high LINC02475 expression with poor overall survival and progression-free survival of HCC patients (Figures 3F,G). Moreover, the positive correlation between LINC02475 expression and poor patients’ prognosis remained significant in the subgroup analyses (Figures 3H–N), including in the subgroup patients of elder age, male gender, stage I, stage III, and grade 1. Collectively, these findings suggested LINC02475 as a potential biomarker in the diagnosis and prognosis evaluation of HCC.

### LINC02475 expression served as an independent prognostic indicator in HCC

**FIGURE 4:**
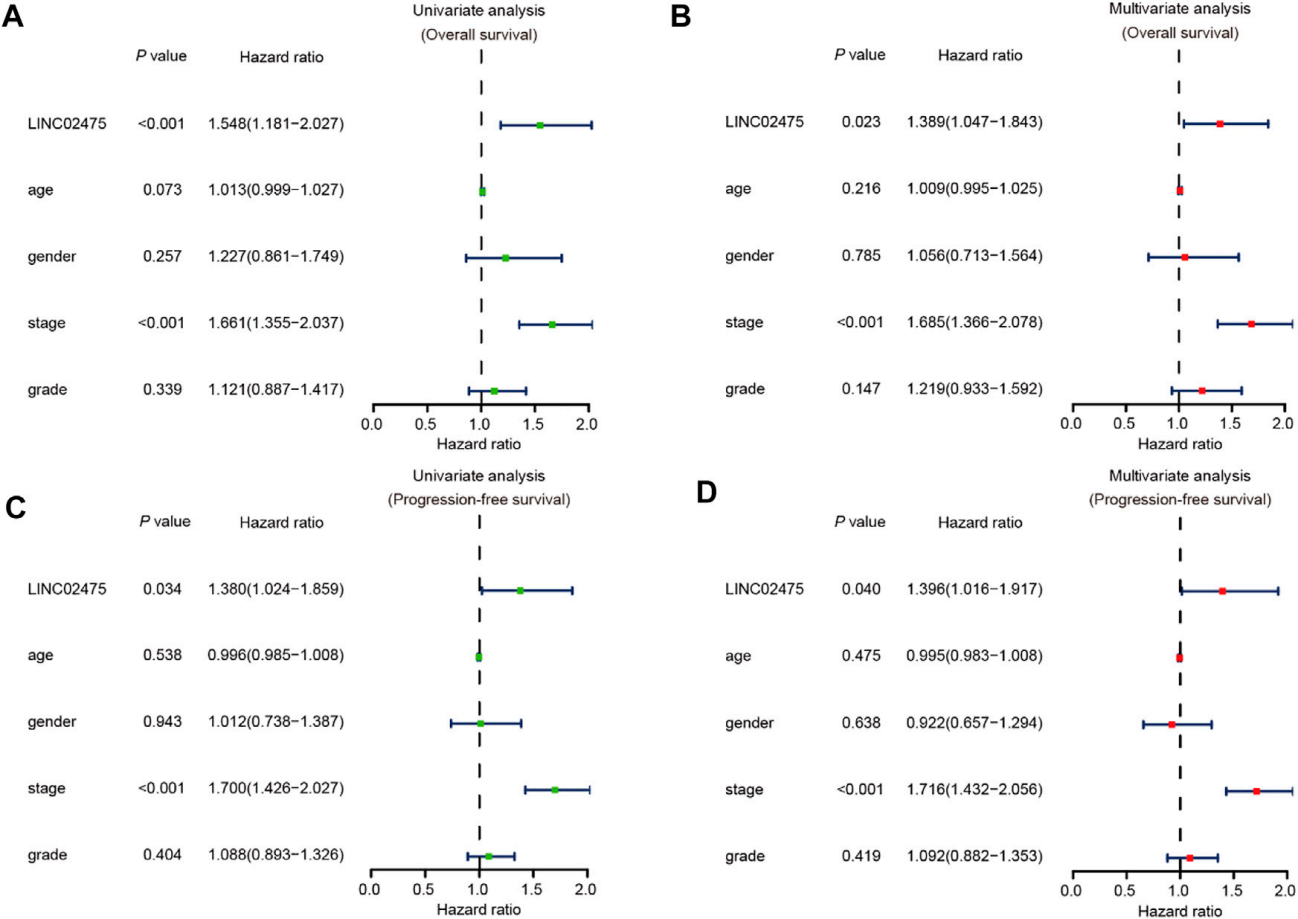
LINC02475 expression serves as an independent prognostic factor in HCC based on TCGA LIHC dataset. **(A,B)** Univariate and multivariate analyses showing the correlation between LINC02475 expression, clinicopathological characteristics, and overall survival of HCC patients. **(C,D)** Univariate and multivariate analyses displaying the association between LINC02475 expression, clinicopathological characteristics, and progression-free survival of HCC patients.

In the subsequent investigation, univariate and multivariate COX hazard analyses were further carried out to reveal the role of LINC02475 expression in HCC. Univariate COX hazard analyses suggested that high LINC02475 expression and cancer stage predicted poor overall survival and progression-free survival of HCC patients. In addition, multivariate COX hazard analyses verified LINC02475 expression as an independent indicator in predicting overall survival (Hazard ratio: 1.389, 95% confidence interval: 1.047–1.843, *p* = 0.023) and progression-free survival of HCC patients (Hazard ratio: 1.396, 95% confidence interval: 1.016–1.917, *p* = 0.040) (Figures 4A–D). Moreover, the cancer stage also served as an independent and unfavorable indicator in HCC, but not other clinical characteristics. These results further demonstrated LINC02475 as a potential biomarker for evaluating the prognosis of HCC patients.

### Silencing LINC02475 inhibited HCC stemness, proliferation, metastasis, radioresistance, and chemoresistance.

**FIGURE 5:**
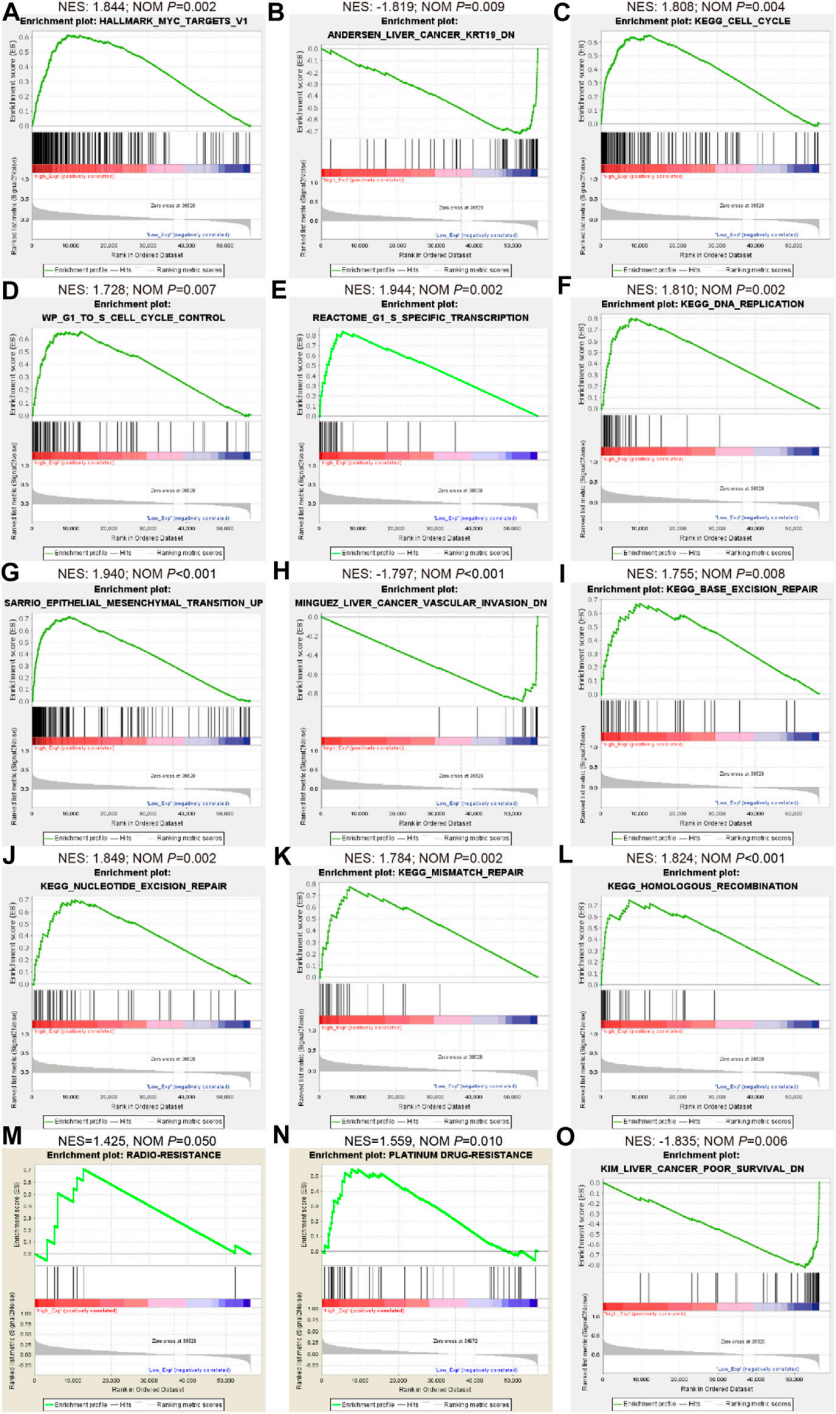
Bioinformatics analyses indicate the involvement of LINC02475 in HCC abnormal signaling pathways and processes as per TCGA database. **(A–O)** GSEA of LINC02475 showing the enriched genesets involved in regulating stemness (Myc and KRT-19) **(A,B)**, proliferation (cell cycle and DNA replication) **(C–F)**, metastasis (EMT and vascular invasion) **(G,H)**, DNA damage repair (nucleotide excision repair, base excision repair, mismatch repair, and homologous recombination) **(I–L)**, radioresistance **(M)**, chemoresistance **(N)**, and poor survival **(O)**-associated pathways and biological processes.

**FIGURE 6:**
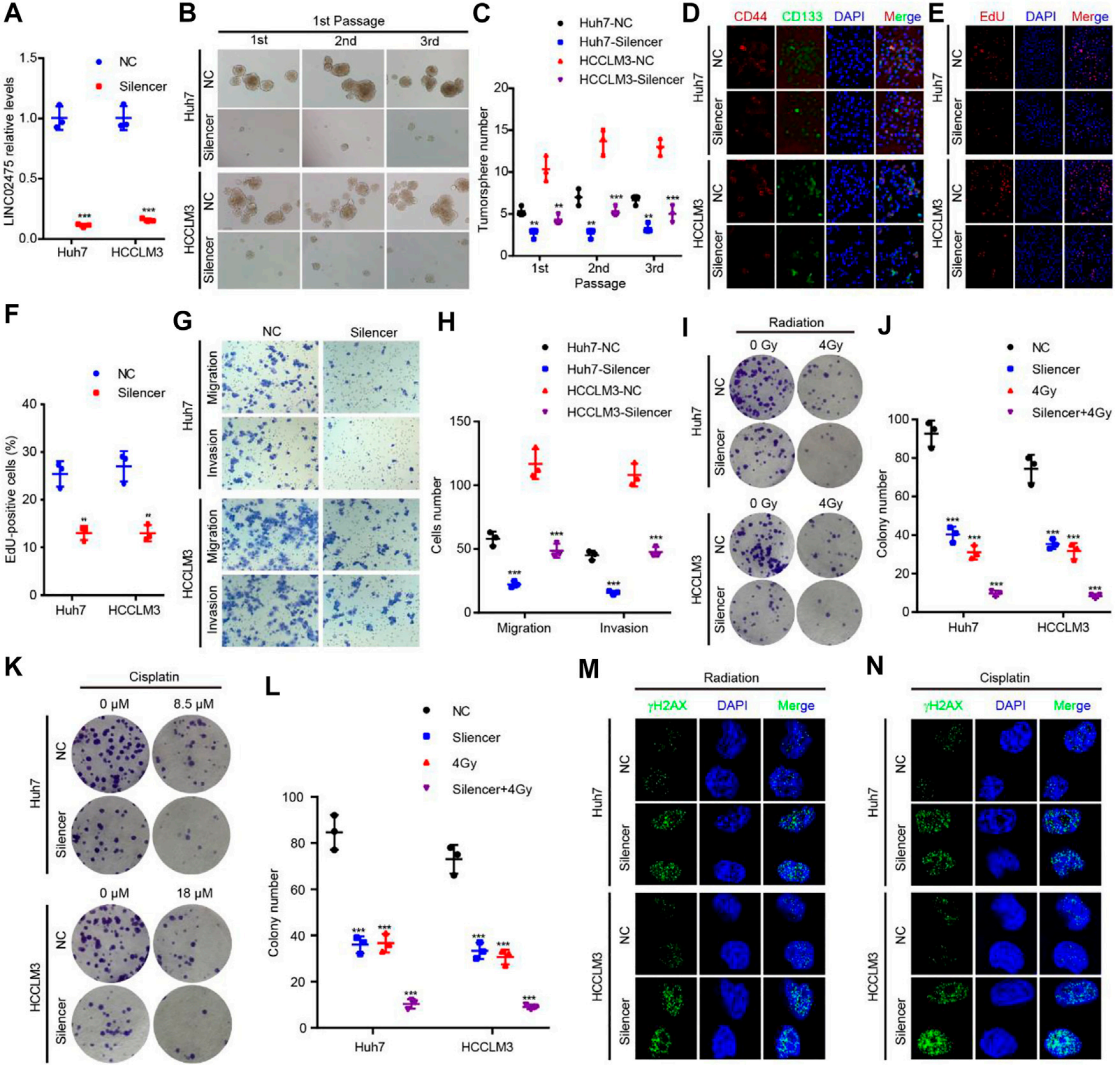
LINC02475 promotes HCC stemness, proliferation, metastasis, radioresistance, and chemoresistance. **(A)** RT-qPCR was used to detect LINC02475 expression in LINC02475-depleted Huh7 and HCCLM3, and the control cells. **(B–L)** Tumorsphere formation **(B,C)**, immunofluorescence **(D)**, EdU **(E,F)**, Transwell **(G,H)**, and colony formation assays **(I–L)** were performed to estimate HCC stemness, proliferation, metastasis, radioresistance, and chemoresistance in LINC02475-silenced Huh7 and HCCLM3, and the control cells. **(M,N)** Immunofluorescence was adopted to measure γ-H2AX (Ser139) expression in LINC02475-depleted Huh7 and HCCLM3, and the control cells. Data are presented as the mean ± SD. **p < 0.01; ***p < 0.001.

Given this confirmation of LINC02475 contribution to HCC progression, we sought to measure the signaling pathways and biological processes affected by LINC02475 dysregulation. Interestingly, GSEA showed that LINC02475 not only participated in the modulation of stemness-related pathways and processes but also regulated proliferation, metastasis, radioresistance, and chemoresistance-related pathways and processes (Figures 5A–O), including Myc signaling, cell cycle, DNA replication, epithelial-mesenchymal transition (EMT), liver cancer vascular invasion, DNA repair, and so on. Then, silencing LINC02475 was performed in Huh7 and HCCLM3 cells with a smart silencer (Figure 6A). Tumorsphere formation assay and immunofluorescence staining of stemness marker CD44 and CD133 verified that LINC02475 knockdown suppressed HCC stemness (Figures 6B–D). Moreover, EdU assay confirmed that LINC02475 depletion inhibited HCC proliferation (Figures 6E,F). In addition, transwell assay validated the suppressive role of LINC02475 depletion in HCC cells (Figures 6G,H). Furthermore, colony formation assays showed that LINC02475 knockdown improved radioresistance and chemoresistance in cisplatin-treated Huh7 and HCCLM3 cells with the concerntration identified in our previous study (Lin et al., 2019) (Figures 6I–L). Subsequently, immunofluorescence staining verified that the expression of proteins associated with DNA damage repair (γ-H2AX) was enhanced in LINC02475-silenced Huh7 and HCCLM3 cells (Figures 6M,N). These findings collectively suggested the stimulatory role of LINC02475 in HCC stemness, proliferation, metastasis, and therapy resistance.

### The relationship between stemness, proliferation, metastasis, and therapy resistance-associated markers and LINC02475 expression.

**FIGURE 7:**
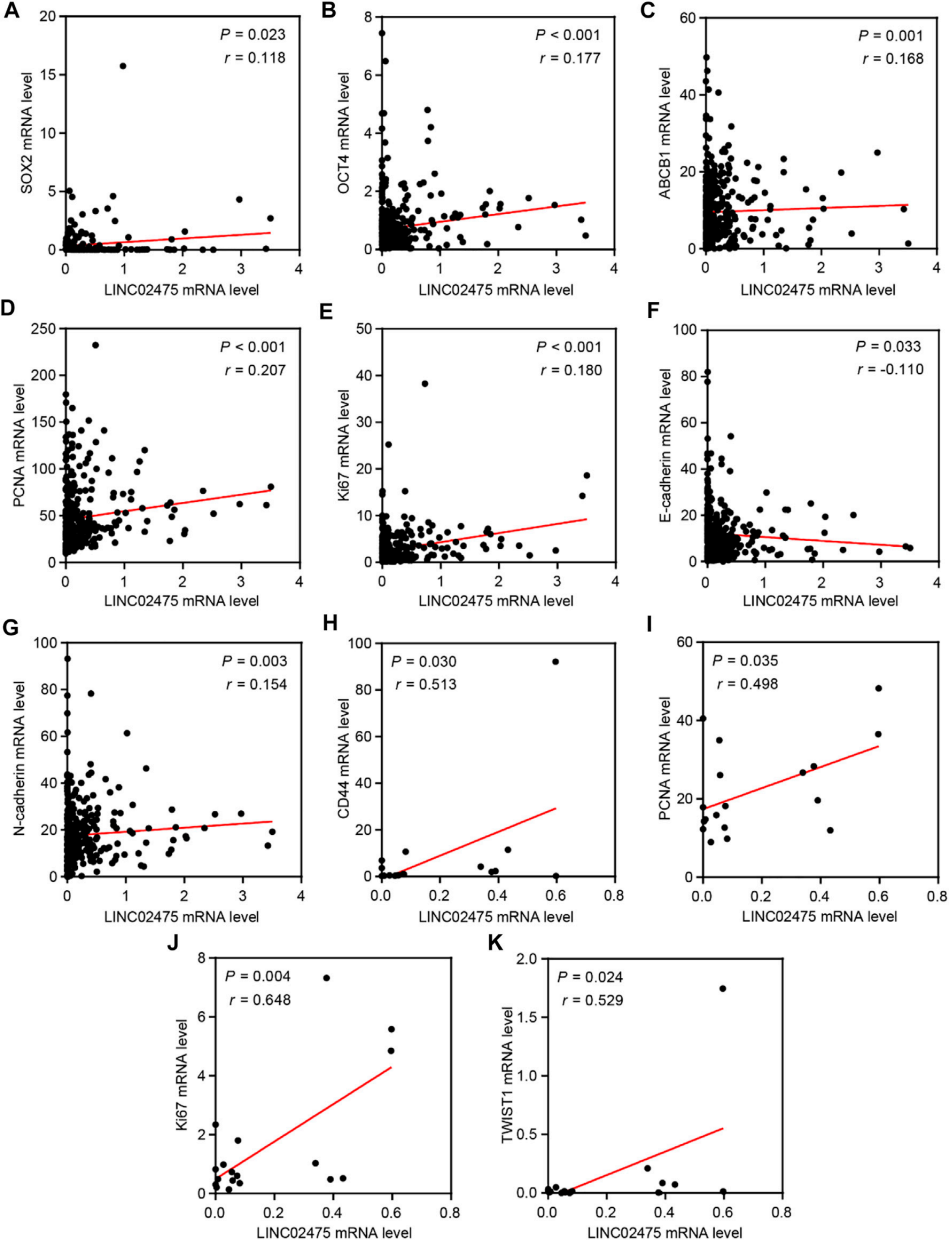
The relationships among LINC02475 and related genes expression. **(A–G)** Relationships among SOX2, OCT4, PCNA, Ki67, E-ca, N-cadherin, ABCB1, and LINC02475 expression in TCGA LICH dataset. **(H–K)** Relationships among CD44, PCNA, Ki67, TWIST1, and LINC02475 expression in ICGC LICA-FR dataset.

In subsequent analyses, we further conducted correlation analyses to identify the relationship between LINC02475 and known oncogenic markers expression. The positive correlations among the expression of SOX2, OCT4, ABCB1, PCNA, Ki67, N-cadherin, and LINC02475, and the negative correlation between E-cadherin and LINC02475 expression was validated in the TCGA LICH database (Figures 7A–G). In addition, the positive correlation among the expression of CD44, PCNA, Ki67, TWIST1, and LINC02475 was verified in the ICGC LICA-FR database (Figures 7H–K). These results consistently demonstrated that LINC02475 functioned as an inducer of HCC progression and a potential biomarker of HCC.

## Discussion

HCC is a severe malignant tumor with high incidence and mortality. Though the existing therapies somehow are beneficial to HCC patients, high recurrence and metastasis rates after therapy are the main challenges to improving the prognosis of HCC (Llovet et al., 2021; Nault and Villanueva, 2021). LncRNAs can serve as biomarkers and potential targets for HCC treatment, ultimately improving the patient’s prognosis (Nault and Villanueva, 2021). Here, we identified a new HCC stemness-related lncRNA (LINC02475) that performed well in HCC diagnosis and independently predicted the poor prognosis of HCC patients. In addition, we elucidated the promotive role of LINC02475 in cell stemness, proliferation, metastasis, radioresistance, and chemoresistance contributing to HCC progression.

In recent years, bioinformatics analysis has played a crucial role in the screening of disease-related targets and the construction of prognostic models in the biomedical field. The unclear function and mechanism of most HCC-related lncRNAs restrict their further development and utilization, and further studies are still needed through bioinformatics screening technologies. Our previous studies successfully adopted mRNAsi as a stem cell index to construct a prognostic model associated with cancer stemness (Lin et al., 2021). In this work, we also used mRNAsi as a stem cell index to search the HCC stemness-related lncRNAs. Moreover, GSEA enrichment analyses of mRNAsi-related genes confirmed their involvement in dysregulated signaling pathways that were tightly connected with CSCs properties, including extracellular matrix organization, nuclear division, growth factor binding, cell cycle, PI3K-AKT signaling, focal adhesion, and so on (Yang et al., 2020). Performing differential, correlation analysis, and WGCNA, we identified lncRNAs correlated with HCC stemness and prognosis based on TCGA LICH dataset. The strategy adopted in the present work was different from those reported in previous studies (Zhang et al., 2020; Cai et al., 2021; Xu et al., 2021), and we therefore discovered LINC02475 as a new cancer stemness-associated candidate oncogene that was correlated with a poor prognosis of HCC and was screened by bioinformatics analysis based on large sample data.

A previous study (Iyer et al., 2015) demonstrated LINC02475 as a liver cancer-associated transcript 1 in the HGNC database (https://www.genenames.org/). Interestingly, LINC02475 was found to be restricted expression toward normal testis as shown in the NCBI database (https://www.ncbi.nlm.nih.gov/). In this work, we further verified that LINC02475 expression was almost absent in normal liver tissues and elevated in HCC. More importantly, we found that LINC02475 expression performed well in the diagnosis of HCC and revealed LINC02475 as an independent prognostic indicator for overall survival and progression-free survival of HCC patients. The existing evidence implies the application of LINC02475 in the precise diagnosis of HCC and accurate prediction of patients’ prognosis, and highlights LINC02475 as a highly selective target providing preferred optimized therapeutic strategies. Moreover, α-fetoprotein (AFP), a fetal serum protein, is the gold standard for surveillance of HCC and occurs mainly in the development of HCC or germ cell tumors (El-Bahrawy, 2010; Tzartzeva et al., 2018), providing more clues that LINC02475 might exhibit a huge advantage as a biomarker like AFP in HCC diagnosis and prognosis evaluation based on the restricted expression of LINC02475 toward normal testis.

CSCs play a crucial role in modulating HCC stemness, proliferation, metastasis, recurrence, as well as therapeutic resistance via epigenetic disruption or signaling pathway dysregulation (Liu et al., 2020; Wang et al., 2021). In this work, GSEA enrichment analyses in low and high LINC02475 groups suggested the participation of LINC02475 in regulating the characteristics of CSCs, including cell stemness, cell cycle and DNA replication, EMT, DNA damage repair that are responsible for cancer cell resistance to radiotherapy and chemotherapy (Lin et al., 2020; Meyer et al., 2020; Zhong and Virshup, 2020). In the subsequent investigations, we validated the biological activities of LINC02475 in augmenting cell stemness, DNA replication, EMT, and DNA damage repair, thus facilitating HCC proliferation, metastasis, radioresistance, and chemoresistance. In addition, the correlation analysis based on TCGA and ICGC database confirmed the relationship between the expression of stemness, DNA replication, EMT, DNA damage repair, and therapy resistance-associated markers and LINC02475.

## Conclusion

**FIGURE 8:**
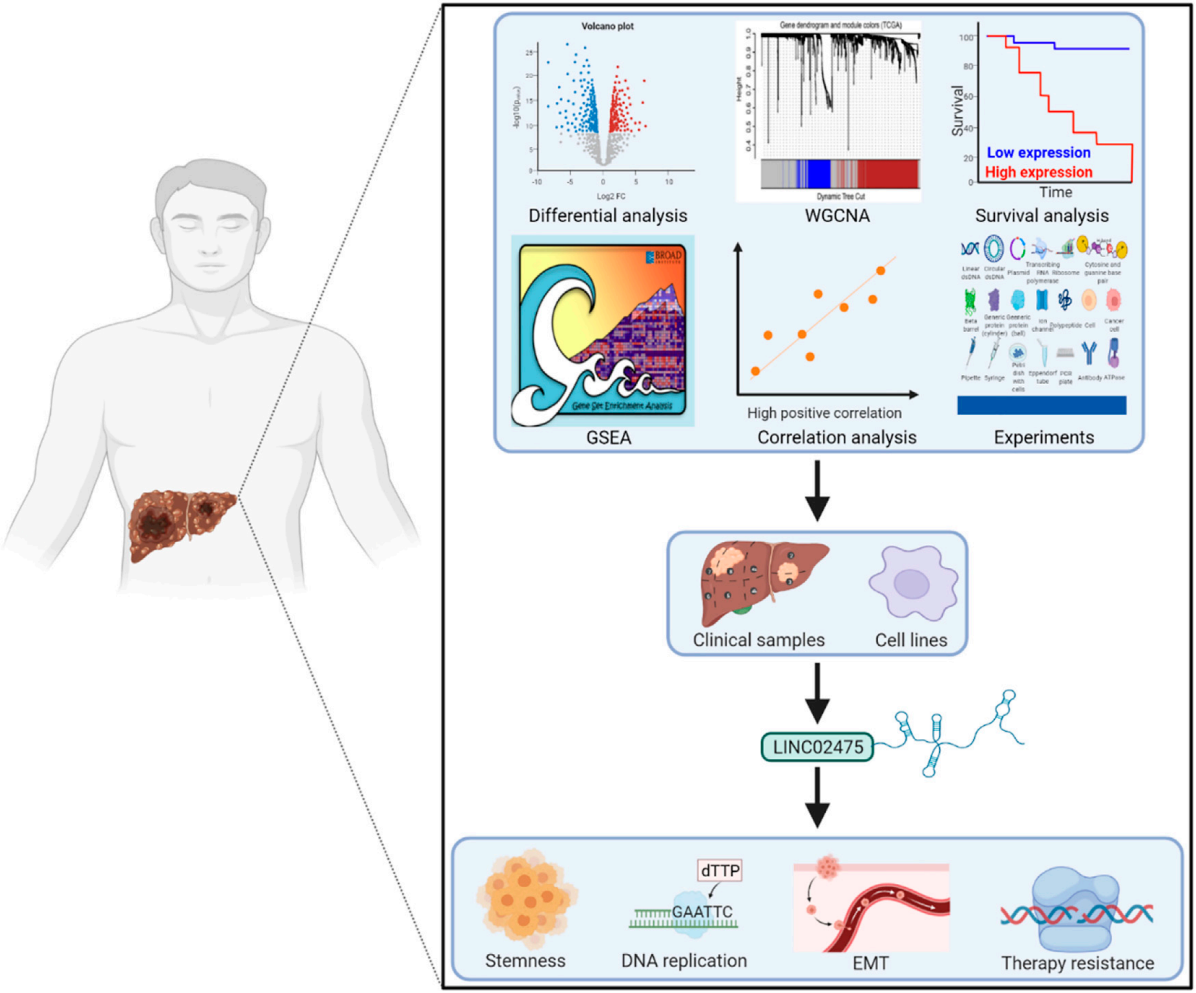
Working model of HCC stemness and prognosis-related LINC02475 in modulating stemness, DNA replication, EMT, and DNA damage repair.

Taken together, cancer stemness-associated LINC02475 performed well in HCC diagnosis, independently predicted a poor prognosis, and promoted HCC progression by inducing stemness, proliferation, metastasis, radioresistance, and chemoresistance (Figure 8). Our study lays a foundation for the clinical translational application of LINC02475 in targeting HCC stem cells and provides LINC02475 as a novel biomarker and target for the diagnosis, the prognosis evaluation, as well as the treatment of HCC.

## Data Availability

The datasets presented in this study can be found in online repositories. The names of the repository/repositories and accession number(s) can be found in the article/Supplementary Material.

## Abbreviations

HCC, Hepatocellular carcinoma; CSCs, Cancer stem cells; TCGA, The Cancer Genome Atlas; ICGC, International Cancer Genome Consortium; LIHC, Liver hepatocellular carcinoma; LICA-FR, Liver Cancer-FR; WGCNA, Weighted gene co-expression network analysis; GSEA, Gene set enrichment analysis; qPCR, Quantitative RT-PCR; mRNAsi, mRNA expression-based stemness index; mDNAsi, DNA methylation-based stemness index; DEGs, Differentially expressed genes; EMT, Epithelial-mesenchymal transition.
