# Supplementary material for: Cancer stemness-associated LINC02475 serves as a novel biomarker for diagnosis and prognosis prediction of hepatocellular carcinoma
Source: Front Genet. 2022 Sep 2;13:991936. doi: 10.3389/fgene.2022.991936 (PMC9479154; doi:10.3389/fgene.2022.991936)
Supplement: Supplementary file 1 [file Table1.docx]

Supplemental file for

**Cancer stemness-associated LINC02475 serves as a novel biomarker for diagnosis and prognosis prediction of hepatocellular carcinoma**

Xian Lin ^1^, Lianxiang Luo ^2, 3^, Yujiao Zou ^4^, Jian Chen ^1^**^,^** ^*^

^1^ Shenzhen Key Laboratory of Inflammatory and Immunology Diseases, Peking University Shenzhen Hospital, Shenzhen Peking University-The Hong Kong University of Science and Technology Medical Center, Shenzhen, 518036, China

^2^ The Marine Biomedical Research Institute, Guangdong Medical University, Zhanjiang, 524023, China.

^3^ The Marine Biomedical Research Institute of Guangdong Zhanjiang, Zhanjiang, 524023, China.

^4^ Department of Radiation oncology, Zhujiang Hospital, Southern Medical University, Guangzhou, 510000, China

* Correspondence:

**Jian Chen:** Shenzhen Key Laboratory of Inflammatory and Immunology Diseases, Peking University Shenzhen Hospital, Shenzhen, 518036, China. Email: [chenjian@jnu.edu.cn](mailto:chenjian@jnu.edu.cn)

**This file includes:**

Supplementary Table S1.

**Supplementary Table S1.** A list of antibodies used for IF

| Antibody | Cat. No | Company | Species | Dilution |
| --- | --- | --- | --- | --- |
| CD44 | 3570 | CST | Mouse | 1:1000 |
| CD133 | 18470-1-AP | Proteintech | Rabbit | 1:1000 |
| γ-H2AX | ab11174 | Abcam | Rabbit | 1:500 |

IF: immunofluorescence
