# Supplementary material for: Cancer stemness-associated LINC02475 serves as a novel biomarker for diagnosis and prognosis prediction of hepatocellular carcinoma
Source: Front Genet. 2022 Sep 2;13:991936. doi: 10.3389/fgene.2022.991936 (PMC9479154; doi:10.3389/fgene.2022.991936)
Supplement: Supplementary file 2 [file Image1.pdf]

Supplemental file for

Cancer stemness-associated LINC02475 serves as a novel biomarker for diagnosis and prognosis prediction of hepatocellular carcinoma

Xian Lin [1], Lianxiang Luo [2,3], Yujiao Zou [4], Jian Chen [1,*]

[1] Shenzhen Key Laboratory of Inflammatory and Immunology Diseases, Peking University Shenzhen Hospital, Shenzhen Peking University-The Hong Kong University of Science and Technology Medical Center, Shenzhen, 518036, China

[2] The Marine Biomedical Research Institute, Guangdong Medical University, Zhanjiang, 524023, China.

[3] The Marine Biomedical Research Institute of Guangdong Zhanjiang, Zhanjiang, 524023, China.

[4] Department of Radiation oncology, Zhujiang Hospital, Southern Medical University, Guangzhou, 510000, China

* Correspondence:

Jian Chen: Shenzhen Key Laboratory of Inflammatory and Immunology Diseases, Peking University Shenzhen Hospital, Shenzhen, 518036, China. Email: chenjian@jnu.edu.cn

This file includes:

Supplementary Figure S1.

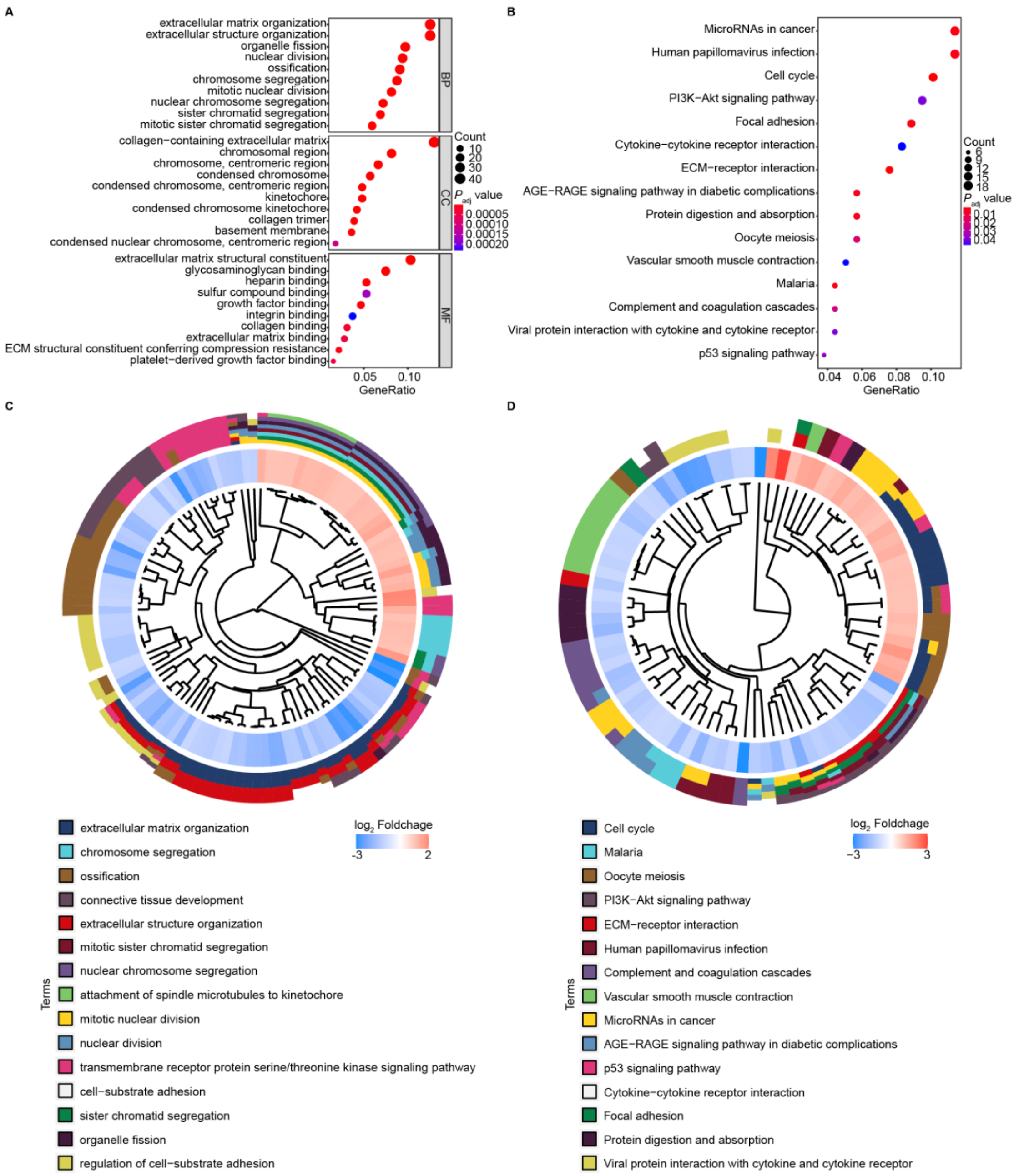

Supplementary Figure S1 Gene set enrichment analysis for the key genes associated with HCC stemness. (**A, B**) The bubble plots showing the top 15 enriched GO and KEGG genesets participating in extracellular matrix organization, nuclear division, growth factor binding, cell cycle, PI3K-AKT signaling, focal adhesion, and so on. BP, biological process; CC, cell component; MF, molecular function. Count: Number of

genes correlated with the enriched GO or KEGG genesets. (**C, D**) The circos displaying the top 15 terms of the enriched GO and KEGG genesets in specific clusters.
